# The Clock Genes *Period 2* and *Cryptochrome 2* Differentially Balance Bone Formation

**DOI:** 10.1371/journal.pone.0011527

**Published:** 2010-07-12

**Authors:** Erik Maronde, Arndt F. Schilling, Sebastian Seitz, Thorsten Schinke, Isabelle Schmutz, Gijsbertus van der Horst, Michael Amling, Urs Albrecht

**Affiliations:** 1 Dr. Senckenbergische Anatomie, Institute for Anatomy III, Goethe University, Frankfurt, Germany; 2 Department of Osteology and Biomechanics, University of Hamburg, Hamburg, Germany; 3 Unit of Biochemistry, Department of Medicine, University of Fribourg, Fribourg, Switzerland; 4 Department of Genetics, Centre for Biomedical Genetics, Erasmus University Medical Centre, Rotterdam, The Netherlands; University of Hong Kong, Hong Kong

## Abstract

**Background:**

Clock genes and their protein products regulate circadian rhythms in mammals but have also been implicated in various physiological processes, including bone formation. Osteoblasts build new mineralized bone whereas osteoclasts degrade it thereby balancing bone formation. To evaluate the contribution of clock components in this process, we investigated mice mutant in clock genes for a bone volume phenotype.

**Methodology/Principal Findings:**

We found that *Per2^Brdm1^* mutant mice as well as mice lacking *Cry2^−/−^* displayed significantly increased bone volume at 12 weeks of age, when bone turnover is high. *Per2^Brdm1^* mutant mice showed alterations in parameters specific for osteoblasts whereas mice lacking *Cry2^−/−^* displayed changes in osteoclast specific parameters. Interestingly, inactivation of both *Per2* and *Cry2* genes leads to normal bone volume as observed in wild type animals. Importantly, osteoclast parameters affected due to the lack of *Cry2*, remained at the level seen in the *Cry2^−/−^* mutants despite the simultaneous inactivation of *Per2*.

**Conclusions/Significance:**

This indicates that *Cry2* and *Per2* affect distinct pathways in the regulation of bone volume with *Cry2* influencing mostly the osteoclastic cellular component of bone and *Per2* acting on osteoblast parameters.

## Introduction

Many biochemical, physiological, and behavioral processes display daily rhythms generated by an internal timekeeping mechanism called the circadian clock. The core oscillator driving this clock is located in the ventral part of the hypothalamus, the suprachiasmatic nuclei (SCN). At the molecular level, this oscillator is thought to be composed of interlocking auto regulatory feedback loops, the transcription/translation feedback loop (TTFL) involving a set of clock genes [1], although other models of how the clock works are increasingly discussed [2], [3]. Among the components driving the mammalian circadian clock are the *Period 1* and *2* (*Per1* and *Per2*) and *Cryptochrome 1* and *2* (*Cry1* and *Cry2*) genes. A mutation in the mouse *Per2* gene (*mPer2*) gene causes a gradual loss of circadian rhythmicity in mice kept in constant darkness (DD; [4] whereas the silencing of *mCry2* leads to immediate arrhythmicity in locomotion behavior [5]. *CRY* proteins are part of the negative limb in the transcriptional/translational feedback loop, whereas PER2 is thought to act positively on Bmal1 expression [6], [7]. Many findings implicated a regulatory effect of CRY proteins on PER2 which was shown in crossed double mutant mice for the *Cry2* deletion being able to compensate at least some of the Per2-related circadian clock defects in *Per2* ^Brdm1^/*Cry2* ^−/−^ double mutants [8]. The proposed functional antagonism of Per2 and Cry2 does not suggest a direct effect of these proteins on each other but that separate sets of genes and functions are affected when Per2 or Cry2 are deleted. Similar effects have been seen before on circadian locomotor behaviour and light-induced phase shift parameters [8].

Studies on the circadian variation of large portions of the genome have been done in bone marrow derived mesenchymal cells and osteoblasts [9]–[11] leading to the proposal that clock genes may be involved in complex processes like bone formation [12].

Beside its fundamental role in circadian physiology *Per2* is involved in regulation of the cell cycle [13], vascular endothelial function [14], addiction behavior [15], food anticipation [16], mood regulation [17], muscle strength [18], LPS-induced interferon gamma production in NK cells [19] and bone density [20]. It appears that expression of *Per* genes in osteoblasts negatively regulates osteoblast proliferation thereby modulating leptin-regulated bone formation [20]. Since expression of most clock genes oscillates in bone we investigated bone volume in mice mutant in clock genes. In particular we were interested in the impact of the *Per2* and *Cry2* genes on bone parameters in single and double mutant female mice and provide genetic evidence for a role of *Cry2* in osteoclast physiology.

## Results

### Bone volume is increased in 3, 12 and 48 week old *Per2^Brdm1^* mice but normal at 24 weeks

**Figure 1:**
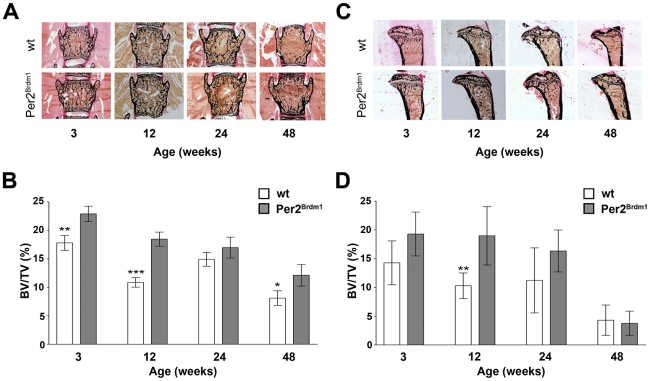
Mineralized bone area in vertebrate spine and tibia and age-dependent differences in bone mineral density in *Per2^Brdm1^* mutant mice. (A) Representative examples of photomicrographs of the mineralized bone area in lumbar vertebrae of 3, 12, 24 and 48 week old *Per2^Brdm1^* mutant mice and their wild type littermates. Black areas represent calcified extracellular matrix, as visualized by Von Kossa staining. (B) Quantitative analysis of BV/TV (bone volume as a ratio of tissue volume) values of lumbar vertebrae for the different age groups. Shown are the means (in percent) ±SD (* = p<0.05, ** = p<0.01, *** = p<0.001, ANOVA with Bonferroni post-test). Note that the significant differences at 3, 12 and 48 weeks of age is absent in the 24 week age group. (C) Representative examples of photomicrographs of the mineralized bone area in tibiae of 3, 12, 24 and 48 week old female wild type and *Per2^Brdm1^* mutant mice. Black areas represent calcified extracellular matrix, as visualized by Von Kossa staining. (D) Quantitative analysis of BV/TV values of tibiae for the different age groups. Shown are the means±SD (* = p<0.05, ** = p<0.01, *** = p<0.001, ANOVA with Bonferroni post-test).

To determine the optimal age to investigate clock gene mediated bone phenotypes we tested bone volume at various ages in *Per2^Brdm1^* mice and compared them with wild type littermates. We found that *Per2^Brdm1^* animals show an age-dependent bone volume phenotype in both lumbar vertebrae and tibiae (Figure 1). At 3, 12 and 48 weeks of age bone density was increased in vertebral spine, but was, as shown before, statistically indistinguishable from wild type at 24 weeks of age ([12]; Figure 1B). Only the 12 week old females had significantly higher tibial bone volume (Figure 1D).

In this study we chose 12 week old mice to minimize influence of the bone deteriorating processes occurring with ageing due to reduced oestrogen levels. At this age we also observed the largest difference between wild type and *Per2^Brdm1^* animals (Figure 1) with a mean mineralized area in lumbar vertebrae (bone volume divided by total volume  =  BV/TV) of 10.9±1.9% for wild type and 18.5±2.8% for *Per2^Brdm1^* mice (p≤0.001; mean±SD). The increased volume in *Per2^Brdm1^* mice involved both cortical and spongiosal structures and was not only present in female, but also in male animals (Figure S3). These findings indicate that *Per2* deficiency leads to overall increased bone volume with age-dependency in female mice.

### Bone formation rate is increased in *Per2^Brdm1^* mice

**Figure 2:**
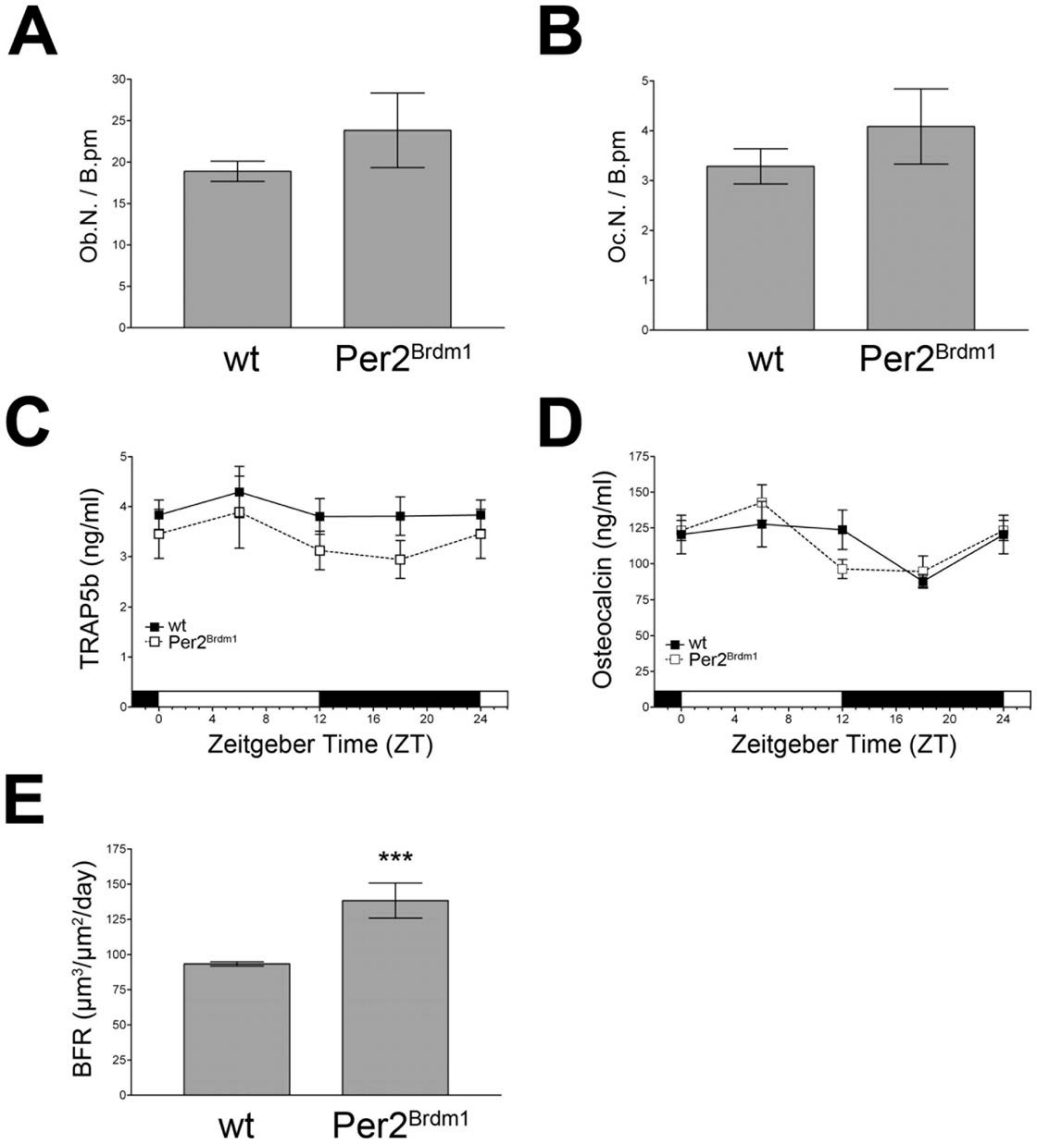
Osteoblast, osteoclast and serum parameters in *Per2^Brdm1^* mutant mice. (A) Osteoblast number per bone perimeter (Ob.N./B.pm) in 12 week old wild type and *Per2^Brdm1^* mutant female mice. The number of osteoblasts per bone perimeter is not significantly different between wild type and *Per2^Brdm1^* mice. (B) Osteoclast number per bone perimeter (Oc.N./B.pm) in 12 week old wild type and *Per2^Brdm1^* mutant female mice. The number of osteoclasts per bone perimeter is not significantly different between wild type and *Per2^Brdm1^* mice. (C) Serum levels of the circulating osteoclast marker TRAP5b in female wild type and *Per2^Brdm1^* mice. (D) Serum levels of the osteoblast activity marker osteocalcin in female wild type and *Per2^Brdm1^* mice. (E) Bone formation rate (BFR) (µm^3^/µm^2^/day)) in female wild type and *Per2^Brdm1^* mice. Shown are the means±SD (panel A, B, E) or SEM (panel C and D) (* = p<0.05, ** = p<0.01, *** = p<0.001, ANOVA with Bonferroni post-test).

To determine the origin of the increased bone mineralization in *Per2^Brdm1^* mice, we next investigated various indicators for osteoblastic or osteoclastic involvement in the constitution of the observed bone phenotypes. We found that the number of osteoblasts and osteoclasts per bone perimeter was not different between wild type and *Per2^Brdm1^* mutant animals (Figure 2A and B, respectively). In line with this finding was the observation that the serum concentrations of the circulating osteoclast activity marker TRAP5b [21], [22] as well as the osteoblast activity marker osteocalcin did not differ between wild type and *Per2^Brdm1^* animals (Figure 2C and D, respectively). Neither conventional curve nor cosinor analysis showed any statistically significant difference in the mesor, acrophase or amplitude of the serum osteocalcin profiles (Figure S1). However, the bone formation rate (BFR), which is mainly influenced by osteoblasts, was significantly higher in *Per2^Brdm1^* mice as compared to wild type littermates (p≤0.01; Figure 2E). These findings suggest that other factors regulating osteoblast activity may be altered in *Per2^Brdm1^* mutant mice. In this context we observed that osteoblasts from *Per2^Brdm1^* mutant mice exerted a significantly higher ability to form bone nodules after 14 days in culture (Figure S5).

### Osteoblast markers are not changed in *Cry2^−/−^* mice

**Figure 3:**
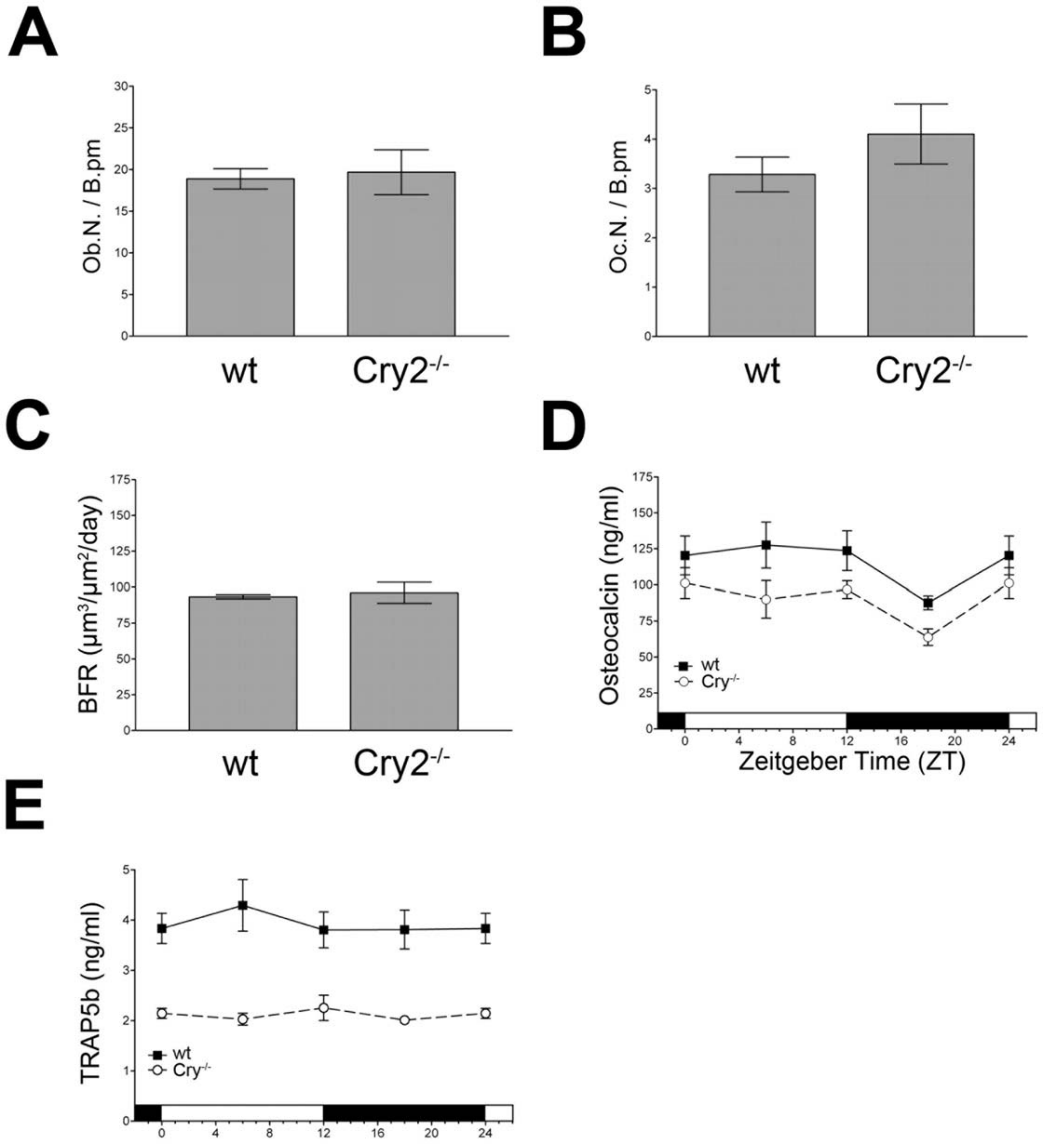
Osteoblast, osteoclast and serum parameters in *Cry2^−/−^* mice. (A) Osteoblast number per bone perimeter (Ob.N./B.pm) in 12 week old wild type and *Cry2^−/−^* mutant mice. (B) Osteoclast number per bone perimeter (Oc.N./B.pm) in 12 week old female wild type and *Cry2^−/−^* mutant mice (C) Bone formation rate (BFR) (µm^3^/µm^2^/day) in female *Cry2^−/−^* mutant mice and wild type controls. (D) Serum levels of the osteoblast activity marker osteocalcin in female wild type or *Cry2^−/−^* mutant mice. (E) Serum levels of the circulating osteoclast marker TRAP5b in 12 week old wild type and *Cry2^−/−^* mutant mice. Shown are the means±SD (panel A–C) or SEM (panel D,E) (* = p<0.05, ** = p<0.01, *** = p<0.001, ANOVA with Bonferroni post-test).

Similar to *Per2^Brdm1^*, also *Cry2^−/−^* mice displayed a mean vertebral spine density (BV/TV) of 21.6±1.5% (mean±SD) in comparison to wild type (10.9±1.9%; Figure 3A). The tibiae of *Cry2^−/−^* mice displayed also a significantly higher bone volume (Figure S4). In order to find a cellular basis of the *Cry2* defect, we investigated the same parameters as described above for the *Per2*-deficient animals. We found that both the number of osteoblasts and the number of osteoclasts per bone perimeter was not different between wild type and *Cry2^−/−^* mutant animals (Figure 3A and B, respectively). Also, bone formation rate and the levels of the osteoblast activity marker osteocalcin in serum did not differ between wild type and *Cry2^−/−^* (ANOVA with Bonferroni post-test; Figure 3C, D, respectively). However, in contrast to *Per2^Brdm1^* mutant mice, the serum levels of the circulating osteoclast activity marker TRAP5b [21], [22] were significantly lowered at any time point in *Cry2^−/−^* animals as compared to wild type mice (p≤0.001; ANOVA with Bonferroni post-test; Figure 3E). This indicates reduced osteoclast activity and hence lower bone resorption in *Cry2^−/−^* mice and correlates with our observation that these animals show higher bone volume. These findings strongly suggest that the bone phenotype of the *Cry2*-deficient female animals is based on lowered osteoclast activity and hence is mechanistically different from the bone phenotype observed in *Per2^Brdm1^* mutant animals.

### Phenotypic compensation in *Per2^Brdm1^/Cry2^−/−^* mice

The two apparently mechanistically different bone phenotypes in *Per2*- or *Cry2*-deficient female mice and the previously described non-allelic compensation of the different chrono-phenotypes that these animals display [8] lead us to investigate bone parameters in the *Per2^Brdm1^*/*Cry2^−/−^* double mutant mice.

**Figure 4:**
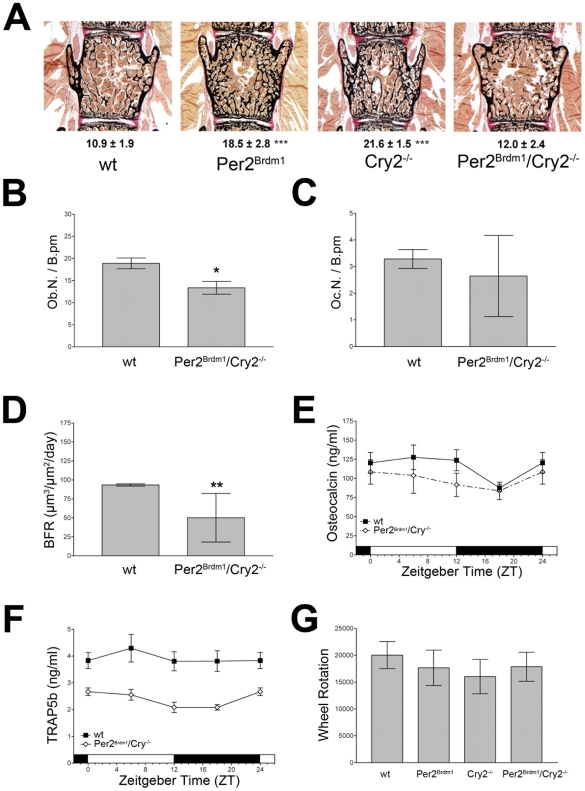
Osteoblast, osteoclast and serum parameters in *Per2Brdm1/Cry2−/−* double mutant mice. A) Representative images of lumbar vertebral spine from 12 week old female Per2Brdm, Cry2−/−, and Per2Brdm1/Cry2−/− mice. B) Osteoblast number per bone perimeter (Ob.N./B.pm) in 12 week old female wild type and Per2Brdm1/Cry2−/− double mutant mice. C) Osteoclast number per bone perimeter (Oc.N./B.pm) in 12 week old wild type and Per2Brdm1/Cry2−/− double mutant mice. D) Bone formation rate (BFR) (µm3/µm2/day) in 12 week old female wild type and Per2Brdm1/Cry2−/− double mutant mice. E) Serum levels of the osteoblast activity marker osteocalcin in wild type and Per2Brdm1/Cry2−/− double mutant animals. F) Serum levels of the circulating osteoclast marker TRAP5b in wild type and Per2Brdm1/Cry2−/− double mutant mice. G) Wheel running activity (total number of wheel rotations evaluated over 5 days under 12∶12 LD) of male wild type, Per2Brdm1, Cry2−/− and Per2Brdm1/Cry2−/− mice. Shown are the means + SD (* = p<0.05, ** = p<0.01, ANOVA with Bonferroni post-test).

In these animals bone volume in vertebrae (BV/TV = 12.4±2.4%) was indistinguishable from wild type (BV/TV = 10.9±1.9%; Figure 4A). Also tibial bone volume was not different between wildtype and *Per2^Brdm1^*/*Cry2^−/−^* double mutant mice (Figure S4). The number of osteoblasts per bone perimeter was significantly lower in double mutant mice (p<0.05; ANOVA with Bonferroni post-test; Figure 4B). Interestingly, the osteoclast number per bone perimeter was normal (Figure 4C) and comparable to *Cry2^−/−^* mice (Figure 3B). These findings correlate well with the reduced bone formation rate (BFR) observed in double mutant mice, as compared to wild type controls (p<0.01; ANOVA with Bonferroni post-test; Figure 4D). Osteocalcin levels indicate normal activity of these osteoblasts (Figure 4E). However, similar to the *Cry2^−/−^* mutants, the circulating osteoclast activity marker TRAP5b was significantly lowered in plasma of double mutant mice at all times (Figure 4F), suggesting lowered osteoclast-dependent bone degradation. It appears that *Per2* affects the osteoblastic parameter bone formation (anabolic process), whereas *Cry2* influences osteoclast activity (catabolic process). Hence, the two clock genes affect bone mineral density in an opposite manner.

### Mechanical load is normal in *Per2^Brdm1^*, *Cry2^−/−^* and *Per2^Brdm1^/Cry2^−/−^* mice

An important parameter influencing bone strength and density is mechanical load. Under constant conditions wheel running activity of all four investigated genotypes was equal (Figure 4F). Moreover, under light/dark conditions *Per2^Brdm1^* and *Cry2^−/−^* mice were as active as wild type and double mutant animals. Another possible influence may be different body weight between the genotypes. Although *Per2^Brdm1^* are slightly heavier than wild type littermates in the first few months of age, they are not different at older age [23], [24]. The body weight of the *Cry2^−/−^* mice did not differ from wildtype. Since *Per2^Brdm1^*/*Cry2^−/−^* mice are also slightly heavier than wild type in the same time period as the *Per2^Brdm1^* mutants it is unlikely that weight is the reason for the observed differences in bone density between genotypes.

## Discussion

*Per2^Brdm1^/Cry2^−/−^* double mutants display wild type bone volume suggesting that *Cry2* can act as a non-allelic suppressor of *Per2* in bone formation. However, *Per2^Brdm1^* animals display increased bone formation and *Cry2^−/−^* mice decreased bone resorption. Therefore, we expected to find a “super-dense-bone” phenotype in the double mutant animals, which is in contrast to what we observed. How can increased bone formation and reduced bone resorption combine to a seemingly normal bone? The answer could lie in the central regulation of bone remodelling that is determined by both afferent and efferent signalling through the hypothalamus.

It has been described that the leptin signal originating in peripheral adipocytes is processed in the hypothalamus from where mediators including neuropeptide Y (NPY) and neuromedin U (NMU) affect osteoblast and osteoclast function [25]. This hypothesis is bolstered by the observation that NMU affects bone remodelling [26]. We observed higher NMU-precursor levels in *Per2^Brdm1^* mutant mice in comparison to wildtype littermates at ZT04 (four hours after lights on; Figure S6). Alterations in NMU levels support the view that hypothalamic mechanisms are responsible for the rescue of the bone phenotype in *Per2^Brdm1^/Cry2^−/−^* double mutant mice. For NMU both systemic [25], [26] and direct cellular [27] influence on osteoblast parameters has been shown making NMU a potential candidate for the mediation of *Per2* effects on bone mineral density. However, whether neuromedin U affects bone formation on the local cellular, the systemic hypothalamic, or both levels, is unclear [25]–[27]. We also observed elevated parathyroid hormone (PTH) levels in the serum of *Per2^Brdm1^* mutant mice at ZT 04 (Figure S7). Finally, osteoblasts cultivated from Per2-luciferase reporter gene mice displayed autonomous circadian oscillation without any input from the central nervous system (Figure S2)[28]. Autonomous circadian cycling of various mesenchymal stem cells from different sources and osteoblasts has been described before [9], [11], [29], [30] and has been recently reviewed [12]. Taking all these findings together it is highly probable that both local bone regulatory processes in the osteoblasts and osteoclasts and the central oscillator in the SCN contribute to bone formation and bone volume regulation.

Another interesting finding is that the *Cry2^−/−^* phenotype appears to be mostly unaffected by the functional *Per2*-deficit of the *Per2^Brdm1^* mutants. In both the single *Cry2^−/−^* and the double mutant *Per2^Brdm1^/Cry2^−/−^* animals circulating TRAP5b levels are significantly reduced. It has been shown that Cry2 deficiency leads to downregulation of NF*k*B-RelA [31]. NF*k*B -RelA promotes osteoclast differentiation by blocking a RANKL- induced JNK pathway [32]. Therefore NF*k*B -RelA deficiency should lead to lower osteoclastic activity which is in line with our observations and might possibly explain the bone resorption phenotype of both the *Cry2^−/−^* and *Per2^Brdm1^/Cry2^−/−^* deficient mice. However, both osteoblast number and bone formation rate are reduced in the *Per2^Brdm1^/Cry2^−/−^* double mutant animals, but unaffected (osteoblast number) and increased (bone formation rate) in the single mutant *Per2^Brdm1^*. Thus the *Cry 2^−/−^* bone phenotype persists irrespectively of the simultaneous absence of the *Per2* gene. This parallels the finding, that there is no obligatory cross-regulation between bone formation and bone resorption [33]–[35]. Whereas Cry2 exerts its effects on the osteoclasts no matter if Per2 is present or not, the osteoblastic effects of Per2 appear to be Cry2-dependent, since in the absence of Cry2 both bone formation rate and osteoblast number are significantly lowered whereas in the presence of Cry2, the *Per2^Brdm1^* mutant mouse bone formation rate was higher than in wildtype littermates. The mechanistic basis for this observation is unclear, but the data speaks for a non-redundancy of the Cry (Cry1 cannot compensate for the absence of Cry2) genes at least for the bone phenotype.

The model that emerged from earlier studies highlighted a role of clock genes (*Per1* and *Per2*) in the leptin-dependent modulation of osteoblast proliferation. Here we extend these findings and report that the action of clock genes in bone remodelling is not limited to osteoblasts, but also involves osteoclast regulation.

Taken together, our findings illustrate that molecular components of the circadian clock mechanism play a critical role in the anabolic and catabolic mechanisms of bone volume regulation. These functions of the clock genes *Per2* and *Cry2* may not depend on circadian regulation phenomena since the observed bone phenotypes occur under diurnal (light dark) conditions. In particular it appears that *Per2* and *Cry2* have opposite effects on bone metabolism via unknown mechanisms regulating osteoblasts and osteoclasts, respectively. Future studies will show whether specific pharmaceutical targeting of *Cry2* or *Per2* can serve as a new therapeutic avenue to treat bone loss conditions such as osteoporosis.

## Materials and Methods

### Mice

The *Per2^Brdm1^*, *Cry2* ^−/−^, and *Per2^ Brdm1^*/*Cry2^−/−^* mice and wild type littermates (all in a hybrid 129Sv/C57BL6 genetic background) used for this study have been described previously [4], [5], [8]. All animal experiments were approved by the Animal Care Facilities of the Hamburg, Frankfurt and Fribourg Universities and performed according to the Declaration of Helsinki. Mice were fed a standard rodent diet and housed in a regular (12 h light/12 h dark) light/dark cycle (12/12LD). The bone phenotype was analyzed at the ages of 3, 12, 24 and 48 weeks. Given the absence of significant sex differences (data not shown), only data from female mice are presented in this manuscript. To assess dynamic histomorphometric indices, mice were given two injections of calcein green 9 and 2 days before animals were sacrificed. At least five mice per group were subjected to histomorphometry and serum analysis to obtain statistically significant results. For the quantification of bone development a total of 80 (20 per age class) female mice of the clock gene mutant animals or wild type controls were screened by radiography.

### Histomorphometry

Skeletons were fixed in 3.7% PBS-buffered formaldehyde for 18 h at 4°C. After 24-h incubation in 70% ethanol, the lumbar vertebral bodies (L3–L5) and one tibia of each mouse were dehydrated in ascending alcohol concentrations and embedded in methylmethacrylate as described previously [36], [37]. Sections of 5 µm were cut in the sagittal plane on a Microtec rotation microtome (Techno-Med, Munich, Germany). These sections were stained by the van Gieson/von Kossa procedure as described [36]. Nonstained sections of 12 µm were used to determine the bone formation rate (BFR in µm^3^/µm^2^/day).

Parameters of static and dynamic histomorphometry were quantified on toluidine blue–stained undecalcified proximal tibia and lumbar vertebral sections of 5 µm. Analysis of bone volume, trabecular number, trabecular spacing, trabecular thickness, and the determination of osteoblast and osteoclast numbers and surface were carried out according to standardized protocols using the Osteo-Measure histomorphometry system (Osteometrics, Atlanta, GA, USA) [37]. Fluorochrome measurements for the determination of bone formation rate were performed on two nonconsecutive 12-µm sections for each animal. Statistical differences between the groups (n≥5) were assessed by ANOVA with subsequent Bonferroni post test using p≤0.05 as the criterion for significance.

### Biochemical assays

Blood was taken from the same mice 4 times every 6 hours (at Zeitgeber time 0/24, 06, 12 and 18; Zeitgeber time 0 is defined as the start of the light period) retro-orbitally. Serum was produced from retro-orbital blood as described before [38]. Serum concentrations of hormones were quantified using antibody-based detection kits (Osteocalcin, from Immutopics, Los Angeles, CA, USA; TRAP5b from IDS, UK).

### Statistical analysis

Data were analysed by GraphPad prism 3.0. Group comparison was done by Stdents t-test, curve comparison by ANOVA with Bonferroni post-test. Possible differences betewwn the genotypes over the course of the day, namely mesor, amplitude and acrophase changes in the osteocalcin and TRAP5b-levels were analyzed by COSINOR analysis as implemented in the MathLab software.

## Supporting Information

Figure S1 Data on osteocalcin levels at different times (ZT00, ZT06, ZT12 and ZT18) in serum of wildtype, *Per2^Brdm1^*, *Cry2^−/−^*, and *Per2^Brdm1^/Cry2^−/−^* mice were subjected to COSINOR analysis. There was no statistical difference in the mesor, acrophase or amplitude of the osteocalcin profiles.
(0.12 MB PDF)

Figure S2 Calvarial osteoblasts from newborn Period2-promoter-luciferase transgenic (mPer2-luc) mice were prepared as described by Kramer et al (2008). The experiment was started by adding fresh medium containing D-luciferin (100 µM) and relative light emission (RLU) was recorded for 68 hours. The cultured osteoblasts oscillate with a approximately 24 hour period.
(0.01 MB PDF)

Figure S3 The vertebrae of male mice were prepared as described in Materials and Methods of the main text. The BV/TV-value of the 12 week old male *Per2^Brdm1^* was significantly higher than that of wildtype littermates (p≤0.05, Student's t-test).
(0.00 MB PDF)

Figure S4 The tibiae of female mice were prepared as described in Materials and Methods of the main text. The BV/TV-value of the 12 week old female *Cry2^−/−^* mice was significantly higher than that of wildtype littermates (p≤0.01, ANOVA with Bonferroni post-test). Wildtype and *Per2^Brdm1^/Cry2^−/−^* mice were not statistically different.
(0.00 MB PDF)

Figure S5 Primary osteoblasts were obtained by sequential collagenase digestion of calvariae from 3-day-old mice. Osteoblast differentiation was induced at 80% confluency in α-MEM containing 10% FBS, 50 µg/ml ascorbic acid, and 10 mM β-glycerophosphate. Analysis of ECM mineralization was determined by Von Kossa staining as described [4] and reveals an accelerated mineralization of *Per2^Brdm1^* derived primary osteoblast cultures compared with wild-type cultures (p≤0.001, Student's t-test).
(0.00 MB PDF)

Figure S6 Neuromedin U (NMU) precursor levels in plasma at ZT04 (four hours after lights on) were determined by SDS-PAGE and Western blotting using affinity-purified rabbit anti-mouse NMU antiserum (1∶1000; Alpha Diagnostics, San Antonio, TX, USA). NMU precursor levels were significantly higher in plasma from *Per2^Brdm1^* compared to wildtype (p≤0.001, Student's t-test).
(0.00 MB PDF)

Figure S7 Intact parathyroid hormone (iPTH) levels in plasma at ZT04 (four hours after lights on) were determined by the mouse intact PTH ELISA kit (Immutopics, San Clemente, CA, USA). Intact PTH levels were significantly higher in plasma from *Per2^Brdm1^* compared to wildtype (p≤0.01, Student's t-test).
(0.00 MB PDF)
