# Supplementary material for: The Clock Genes Period 2 and Cryptochrome 2 Differentially Balance Bone Formation
Source: PLoS One. 2010 Jul 12;5(7):e11527. doi: 10.1371/journal.pone.0011527 (PMC2902506; doi:10.1371/journal.pone.0011527)

Figure S1

Cosinor analysis

Osteocalcin	wildtype	Per^{Brdm1}	Cry2$^{-/-}$	Per^{Brdm1}/Cry2$^{-/-}$
MESOR	115	114.25	88	97.25
AMPLITUDE	20.099	27.536	13.152	13.124
ACROPHASE	13.38	11.042	12.416	10.309
p-VALUE	0.441	0.237	0.763	0.33

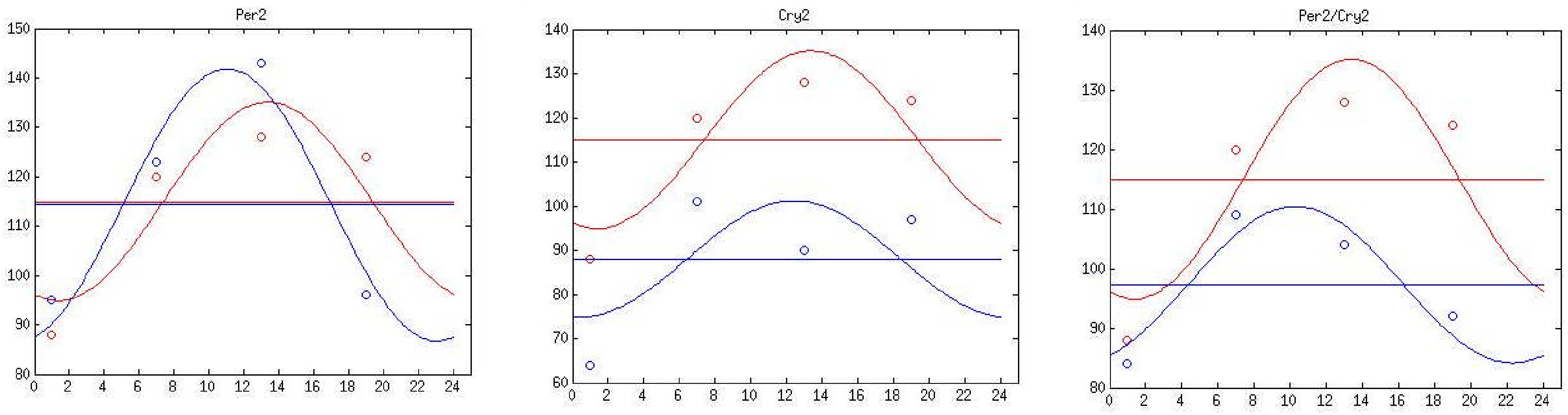

Supplement: Figure S1 — Data on osteocalcin levels at different times (ZT00, ZT06, ZT12 and ZT18) in serum of wildtype, Per2Brdm1, Cry2−/−, and Per2Brdm1/Cry2−/− mice were subjected to COSINOR analysis. There was no statistical difference in the mesor, acrophase or amplitude of the osteocalcin profiles. (0.12 MB PDF) [file pone.0011527.s001.pdf]
