# Supplementary figures and images for: The Clock Genes Period 2 and Cryptochrome 2 Differentially Balance Bone Formation
Source: PLoS One. 2010 Jul 12;5(7):e11527. doi: 10.1371/journal.pone.0011527 (PMC2902506; doi:10.1371/journal.pone.0011527)

Figure S2

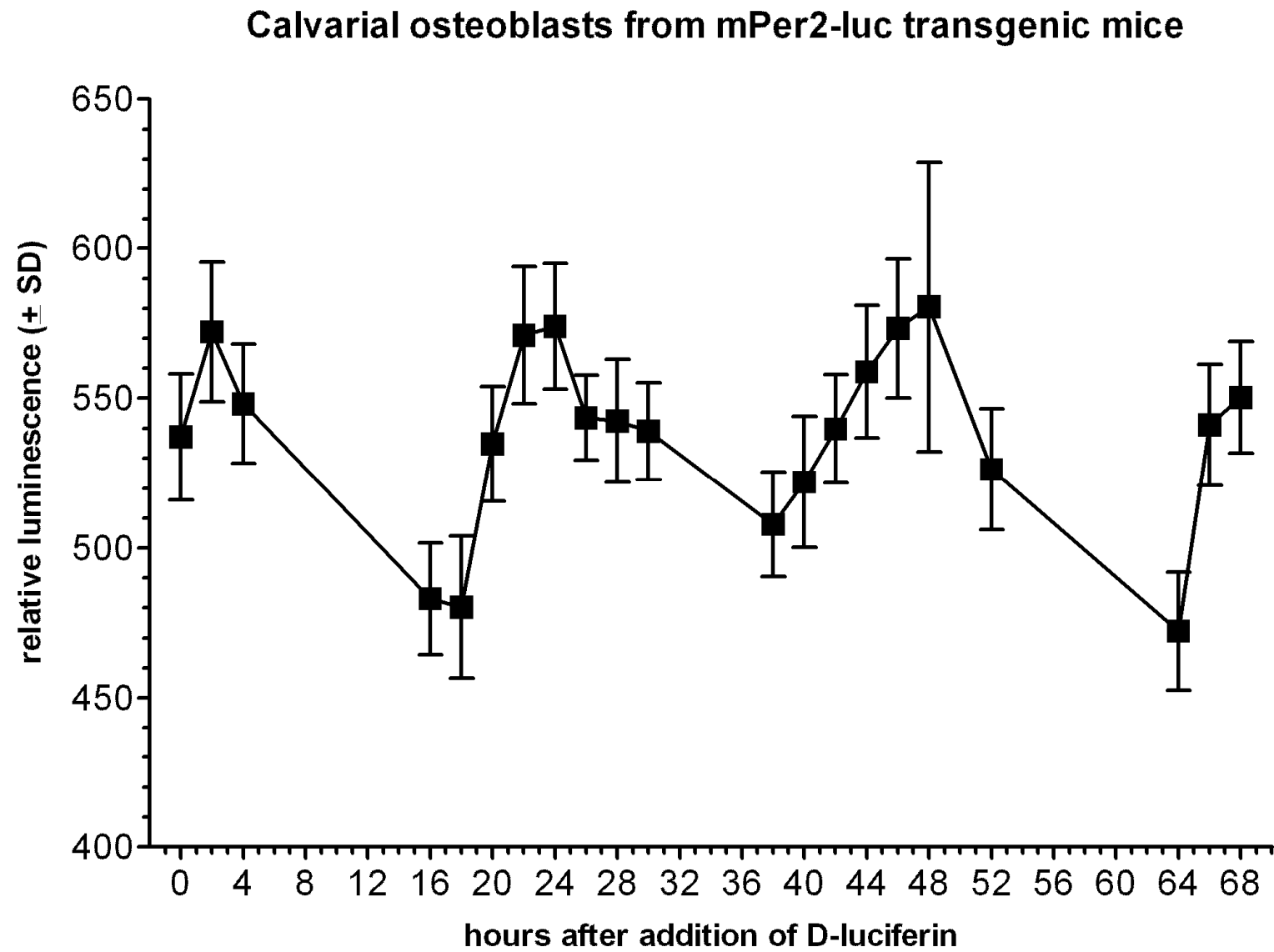

Supplement: Figure S2 — Calvarial osteoblasts from newborn Period2-promoter-luciferase transgenic (mPer2-luc) mice were prepared as described by Kramer et al (2008). The experiment was started by adding fresh medium containing D-luciferin (100 µM) and relative light emission (RLU) was recorded for 68 hours. The cultured osteoblasts oscillate with a approximately 24 hour period. (0.01 MB PDF) [file pone.0011527.s002.pdf]

Figure S3

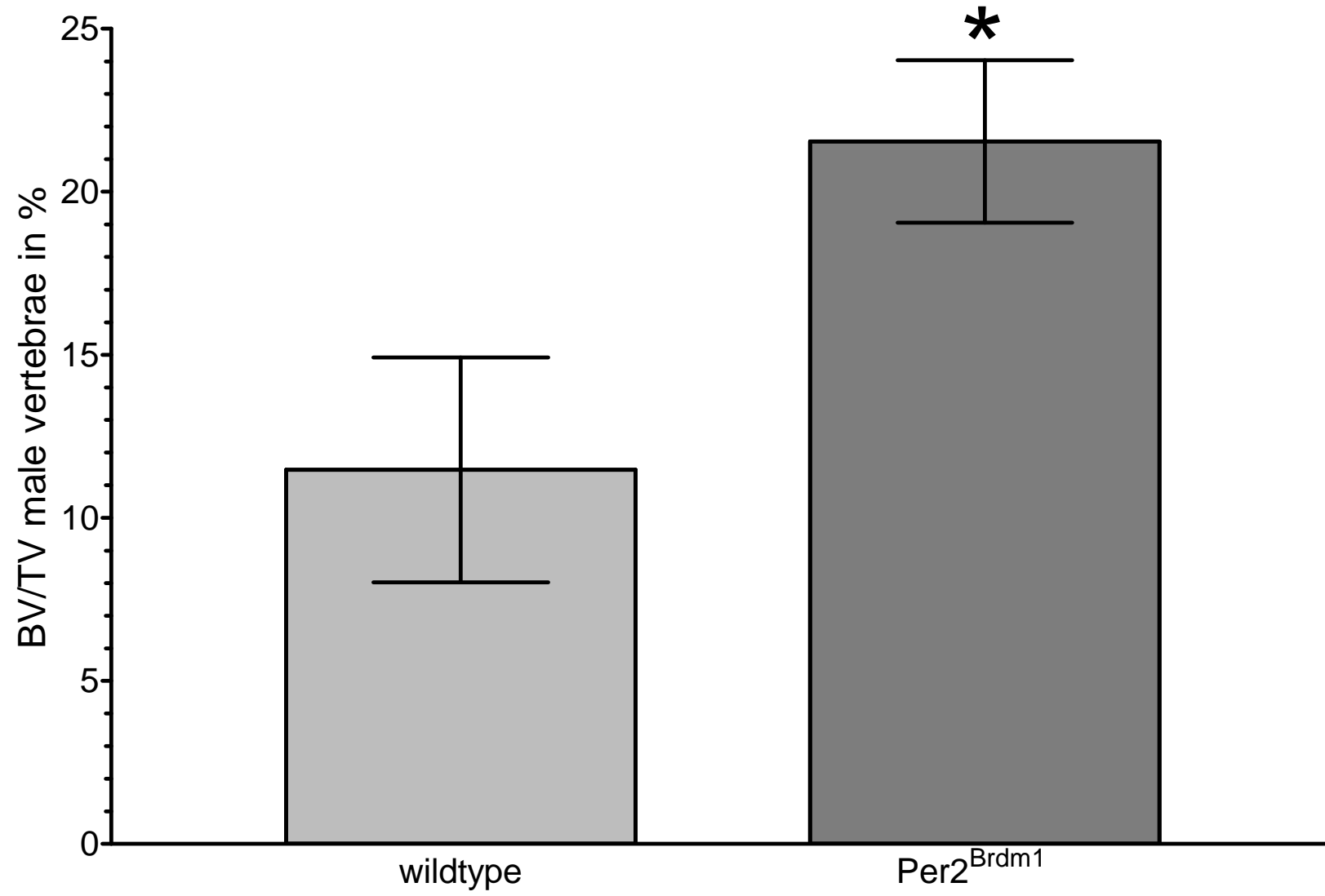

Supplement: Figure S3 — The vertebrae of male mice were prepared as described in Materials and Methods of the main text. The BV/TV-value of the 12 week old male Per2Brdm1 was significantly higher than that of wildtype littermates (p≤0.05, Student's t-test). (0.00 MB PDF) [file pone.0011527.s003.pdf]

Figure S4

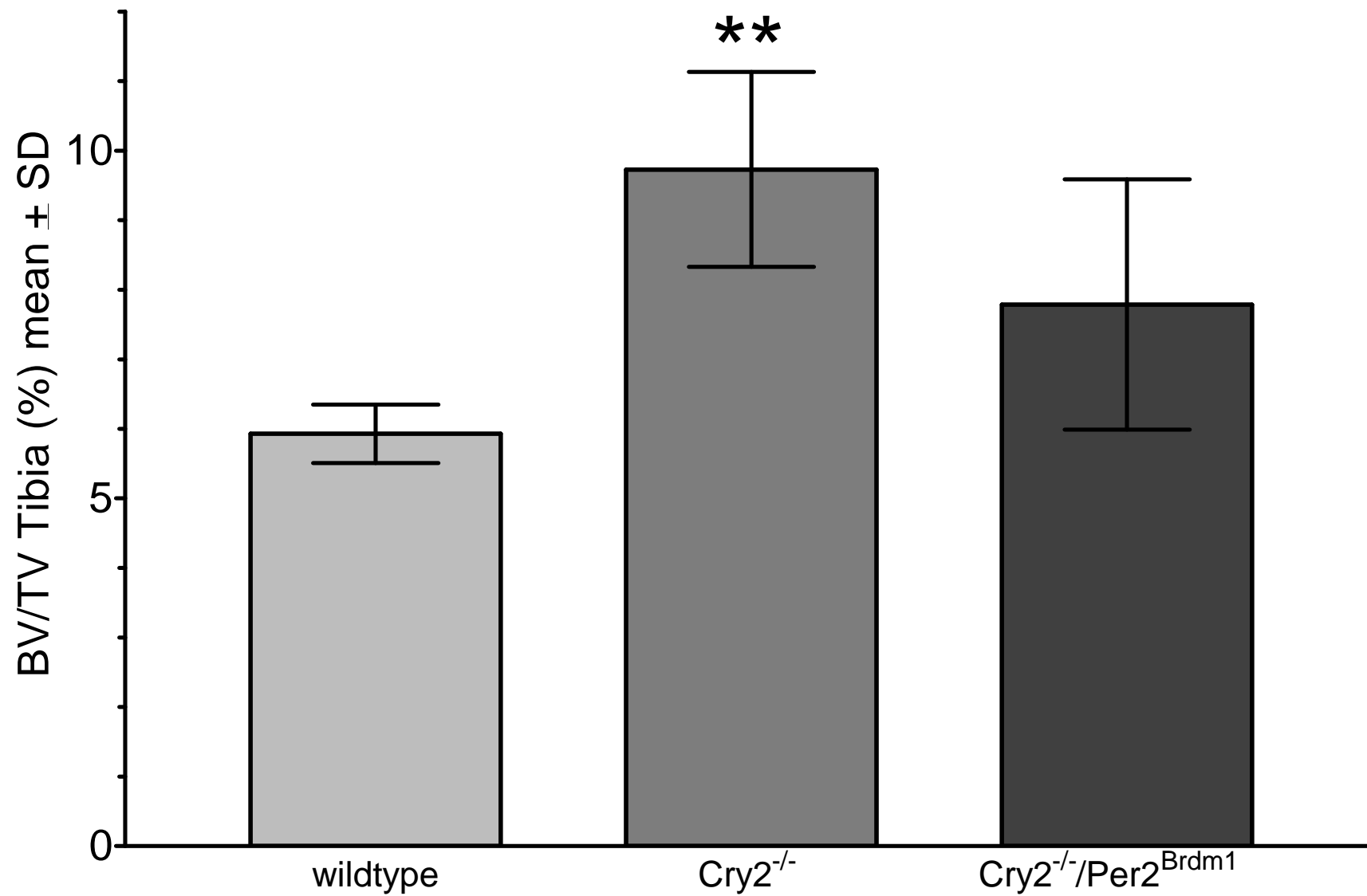

Supplement: Figure S4 — The tibiae of female mice were prepared as described in Materials and Methods of the main text. The BV/TV-value of the 12 week old female Cry2−/− mice was significantly higher than that of wildtype littermates (p≤0.01, ANOVA with Bonferroni post-test). Wildtype and Per2Brdm1/Cry2−/− mice were not statistically different. (0.00 MB PDF) [file pone.0011527.s004.pdf]

Figure S5

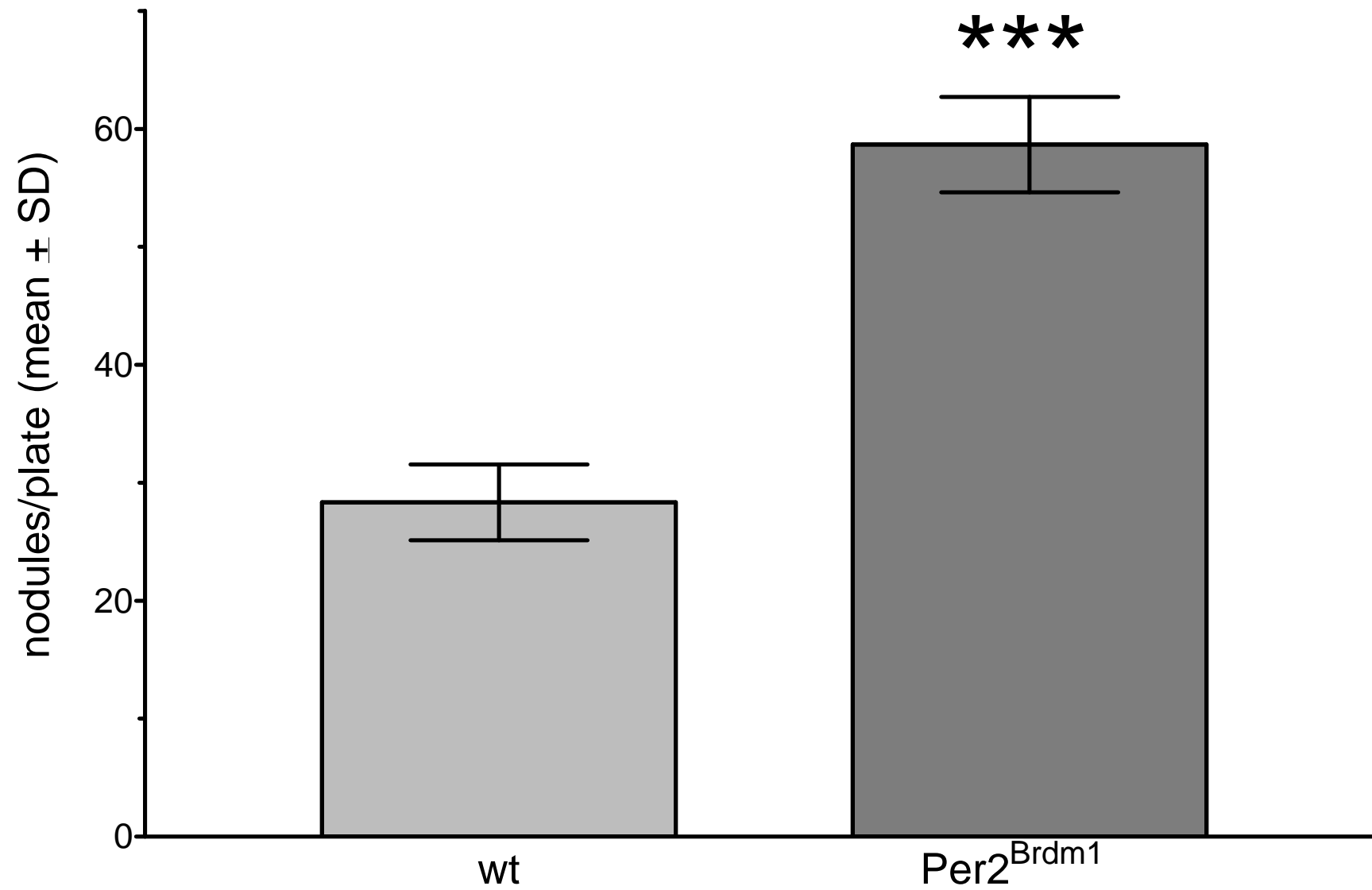

Supplement: Figure S5 — Primary osteoblasts were obtained by sequential collagenase digestion of calvariae from 3-day-old mice. Osteoblast differentiation was induced at 80% confluency in α-MEM containing 10% FBS, 50 µg/ml ascorbic acid, and 10 mM β-glycerophosphate. Analysis of ECM mineralization was determined by Von Kossa staining as described [4] and reveals an accelerated mineralization of Per2Brdm1 derived primary osteoblast cultures compared with wild-type cultures (p≤0.001, Student's t-test). (0.00 MB PDF) [file pone.0011527.s005.pdf]

Figure S6

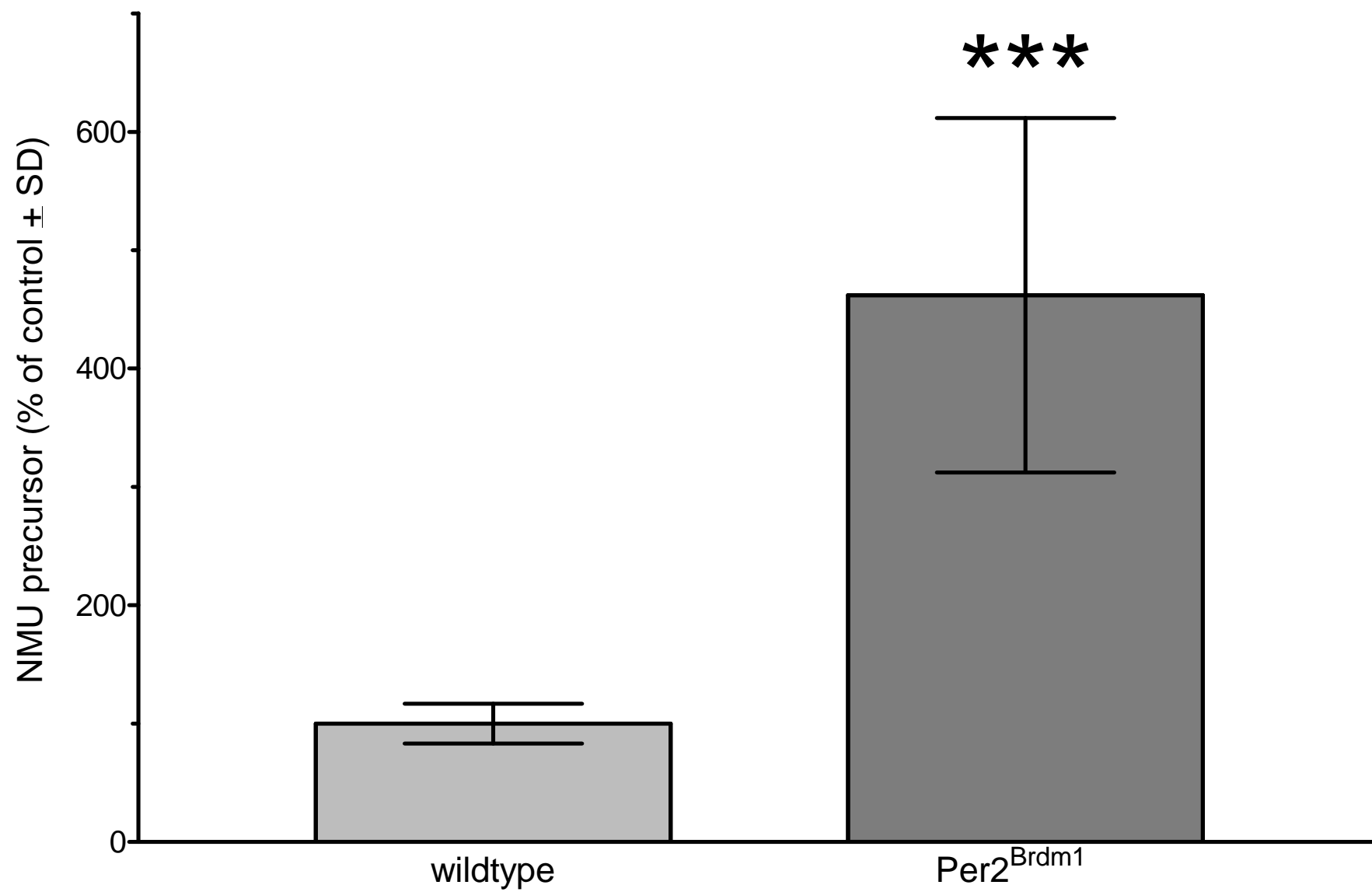

Supplement: Figure S6 — Neuromedin U (NMU) precursor levels in plasma at ZT04 (four hours after lights on) were determined by SDS-PAGE and Western blotting using affinity-purified rabbit anti-mouse NMU antiserum (1∶1000; Alpha Diagnostics, San Antonio, TX, USA). NMU precursor levels were significantly higher in plasma from Per2Brdm1 compared to wildtype (p≤0.001, Student's t-test). (0.00 MB PDF) [file pone.0011527.s006.pdf]

Figure S7

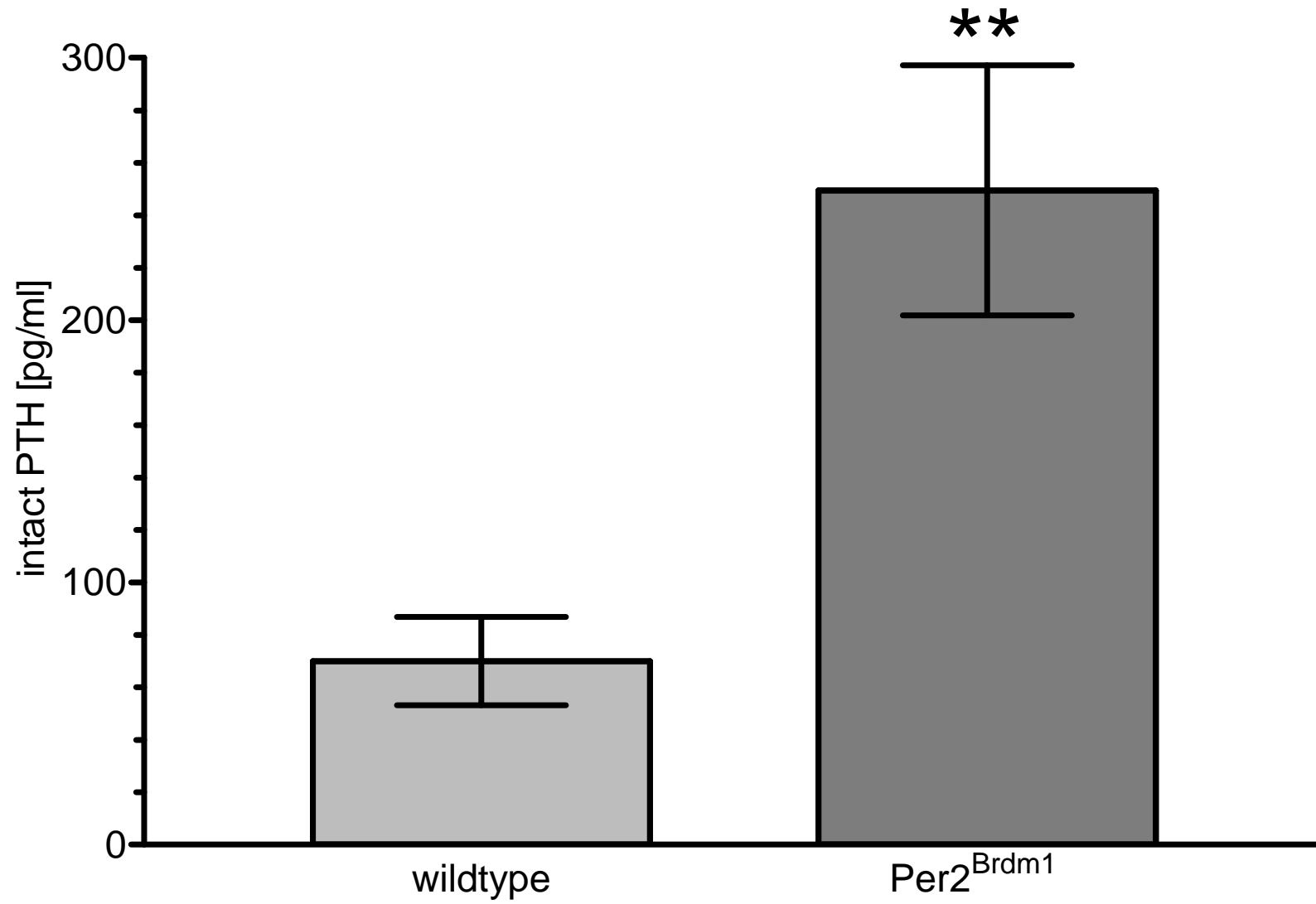

Supplement: Figure S7 — Intact parathyroid hormone (iPTH) levels in plasma at ZT04 (four hours after lights on) were determined by the mouse intact PTH ELISA kit (Immutopics, San Clemente, CA, USA). Intact PTH levels were significantly higher in plasma from Per2Brdm1 compared to wildtype (p≤0.01, Student's t-test). (0.00 MB PDF) [file pone.0011527.s007.pdf]
